# Conservative Treatment of Symptomatic Pudendal Nerve Entrapment Secondary to Sacrotuberous Ligament Ossification

**DOI:** 10.7759/cureus.109925

**Published:** 2026-05-30

**Authors:** Christos Lyrtzis, Apostolos Prinos, Christos Tsiantas, Georgios Trikoilis, Irene Asouhidou, George Paraskevas

**Affiliations:** 1 Department of Anatomy and Surgical Anatomy, Aristotle University of Thessaloniki, Thessaloniki, GRC; 2 Department of Anatomy and Surgical Anatomy, Aristotle University of Thessaloniki, thessaloniki, GRC

**Keywords:** anatomical variation, nerve entrapment, pelvic pain, pudendal neuralgia, sacrotuberous ligament ossification

## Abstract

Sacrotuberous ligament (STL) ossification is an exceptionally rare condition that may lead to pudendal nerve entrapment through mechanical compression within the pelvic region. Pudendal nerve entrapment is frequently underdiagnosed despite causing significant neuropathic symptoms and functional impairment. We present a case of pudendal nerve entrapment secondary to STL ossification that was successfully managed conservatively. A 50-year-old male patient presented with chronic right-sided hip and perineal pain associated with burning sensation, foreign body sensation, sensory disturbances, and erectile dysfunction. Symptoms were aggravated by prolonged sitting and partially relieved by standing. Physical examination demonstrated reduced superficial sensation in the perineal region. Pelvic radiography revealed partial ossification of the right sacrotuberous ligament, while three-dimensional computed tomography confirmed the extent and morphology of the ossified structure. No additional pelvic or spinal pathology was identified. The patient was treated conservatively with ultrasound-guided corticosteroid injections administered at three-month intervals over a one-year period, combined with activity modification and specialized seating support. Progressive clinical improvement was observed, with complete resolution of pain, sensory symptoms, and erectile dysfunction. At two-year follow-up, the patient remained asymptomatic without evidence of recurrence. This case highlights STL ossification as a rare but clinically important cause of pudendal nerve entrapment and chronic perineal pain. Careful clinical assessment and appropriate imaging are essential for accurate diagnosis. Although surgical treatment has been described in isolated cases, conservative management may provide substantial symptomatic relief and should be considered a first-line therapeutic approach in selected patients.

## Introduction

The sacrotuberous ligament (STL) is a major pelvic ligament that contributes significantly to the stability of the sacroiliac region. It originates from the posterior superior iliac spine, posterior inferior iliac spine, lateral sacrum, and upper coccyx, and courses obliquely downward and laterally to insert onto the ischial tuberosity [1]. Along its course, it lies posterior to the sacrospinous ligament, another important stabilizing structure of the pelvis.

The pudendal nerve, arising from the ventral rami of S2-S4, exits the pelvis through the greater sciatic foramen, courses posterior to the sacrospinous ligament and medial to the sacrotuberous ligament, and subsequently re-enters the pelvis through the lesser sciatic foramen before traversing Alcock's canal. Distally, it terminates into three principal branches: the inferior rectal nerve, the perineal nerve, and the dorsal nerve of the penis or clitoris [2]. Functionally, the pudendal nerve provides the principal sensory innervation to the external genitalia, perineum, and perianal region, motor innervation to the external urethral and anal sphincters as well as several pelvic floor muscles, and proprioceptive fibers contributing to continence and normal pelvic floor function [2].

Ossification of the sacrotuberous ligament is an exceedingly rare condition, with only 17 cases reported in the literature to date, all occurring in male patients [1,3]. Given the ligament's important biomechanical role in pelvic stability, ossification may alter the surrounding anatomy and contribute to pelvic dysfunction [3]. In particular, ossification may result in narrowing of the anatomical space traversed by the pudendal nerve, potentially leading to pudendal nerve entrapment.

Pudendal nerve entrapment remains an underdiagnosed clinical entity despite its potentially debilitating symptoms. Patients commonly present with chronic perineal pain, hip pain, burning sensations, or discomfort that is exacerbated by sitting and relieved by standing [4]. Additional manifestations may include sensory disturbances, fecal incontinence, and sexual dysfunction [3].

Management strategies vary depending on symptom severity and the underlying etiology. Although surgical treatment has been described in previously reported cases of sacrotuberous ligament ossification, conservative management approaches have rarely been documented. Surgical excision of the ossified sacrotuberous ligament has been reported to provide symptomatic relief in selected patients with severe or refractory pudendal nerve entrapment, but published experience remains limited to isolated case reports, and concerns persist regarding potential postoperative pelvic instability and secondary biomechanical complications following ligament resection [4]. In this report, we present a case of pudendal nerve entrapment secondary to sacrotuberous ligament ossification that was successfully managed conservatively.

## Case presentation

A 50-year-old male patient engaged in non-manual intellectual work presented with chronic right-sided hip pain of several months' duration. The pain was described as a burning sensation associated with a foreign body sensation and was accompanied by erectile dysfunction. Symptoms were exacerbated in the sitting position and partially relieved upon standing. The patient denied any history of trauma or previous pelvic or spinal surgery. The patient did not report participation in regular high-intensity physical activity, cycling, prolonged running, or other repetitive pelvic-loading exercises.

**Figure 1:**
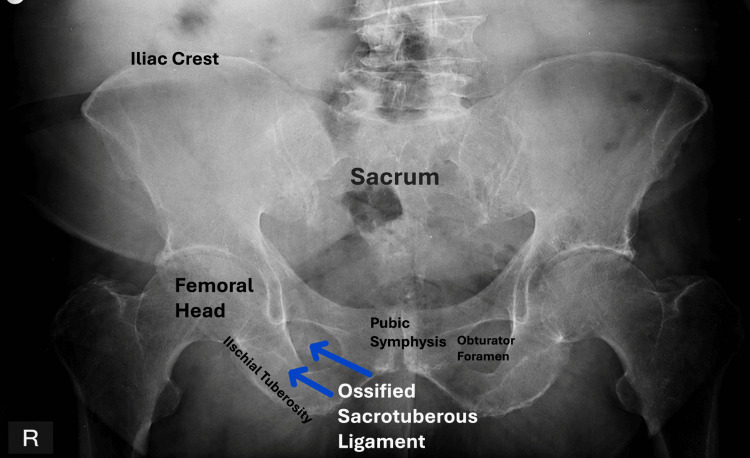
Anteroposterior pelvic radiograph Partial ossification of the right sacrotuberous ligament is demonstrated (arrows), appearing as a fan-shaped ossified structure within the right posterior pelvis adjacent to the ischiopubic ramus. Key anatomical landmarks demonstrated include the sacrum, right iliac crest, right femoral head, right ischial tuberosity, left obturator foramen, and pubic symphysis.

On physical examination, reduced superficial sensation was noted in the perineal region, raising suspicion of pudendal neuropathy. Pelvic radiography demonstrated partial ossification of the right sacrotuberous ligament, appearing as a fan-shaped ossified structure located in the posterior pelvis and extending from the iliac tuberosity (Figure 1).

**Figure 2:**
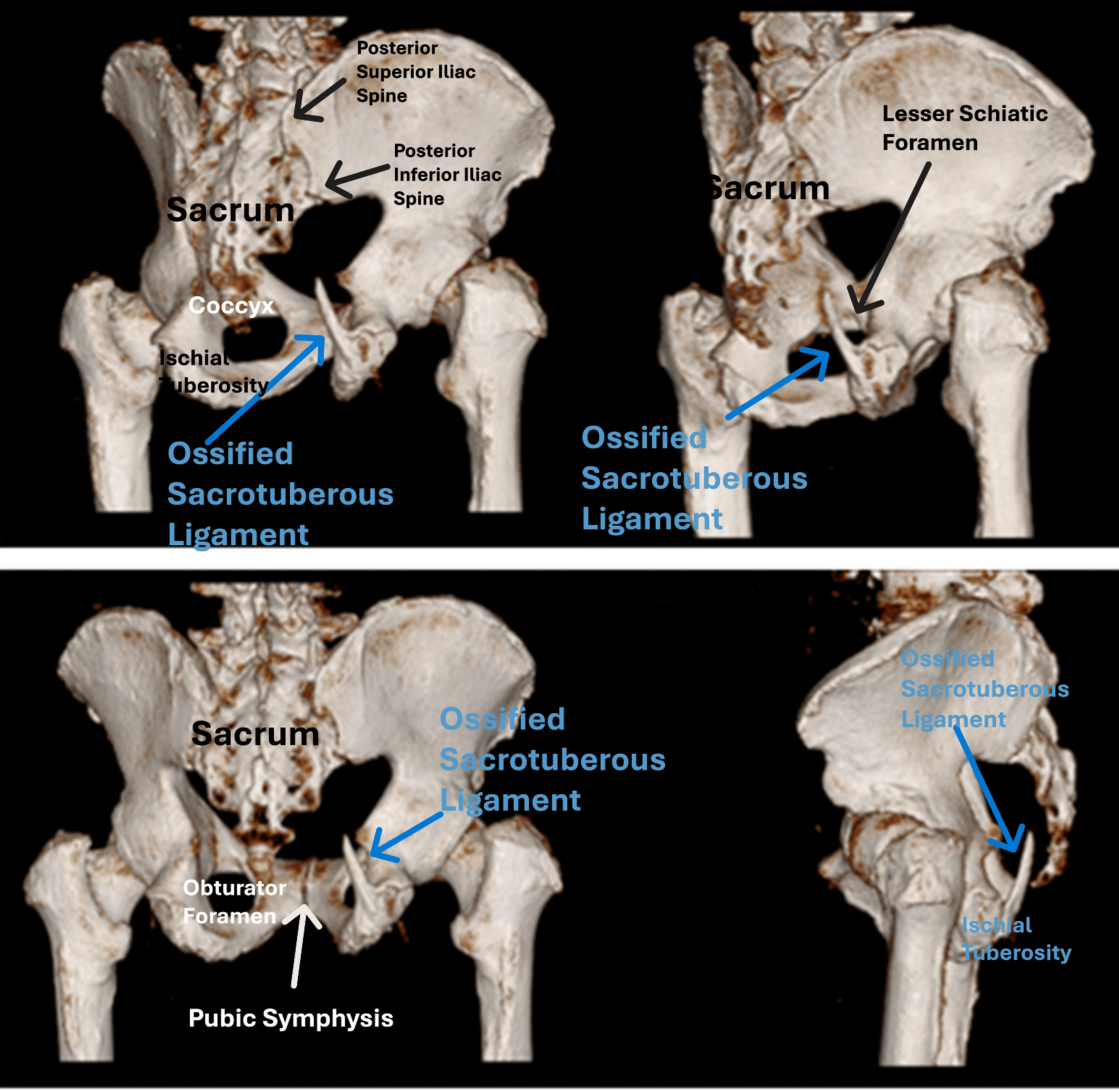
Three-dimensional computed tomography (3D-CT) reconstructions of the pelvis demonstrating partial ossification of the right sacrotuberous ligament The ossified ligament appears as an elongated, fan-shaped calcified structure (arrows) extending from the posterior pelvis toward the ischial region, in close anatomical relation to the expected course of the pudendal nerve. Key anatomical landmarks related to the attachment and course of the ossified sacrotuberous ligament are demonstrated, including the sacrum, posterior superior iliac spine, posterior inferior iliac spine, coccyx, ischial tuberosity, lesser sciatic foramen, obturator foramen, and pubic symphysis.

Subsequent three-dimensional computed tomography (3D-CT) of the pelvis provided clearer delineation of the ossified sacrotuberous ligament and confirmed the radiographic findings (Figure 2). No additional pelvic or spinal pathology was identified.

**Figure 3:**
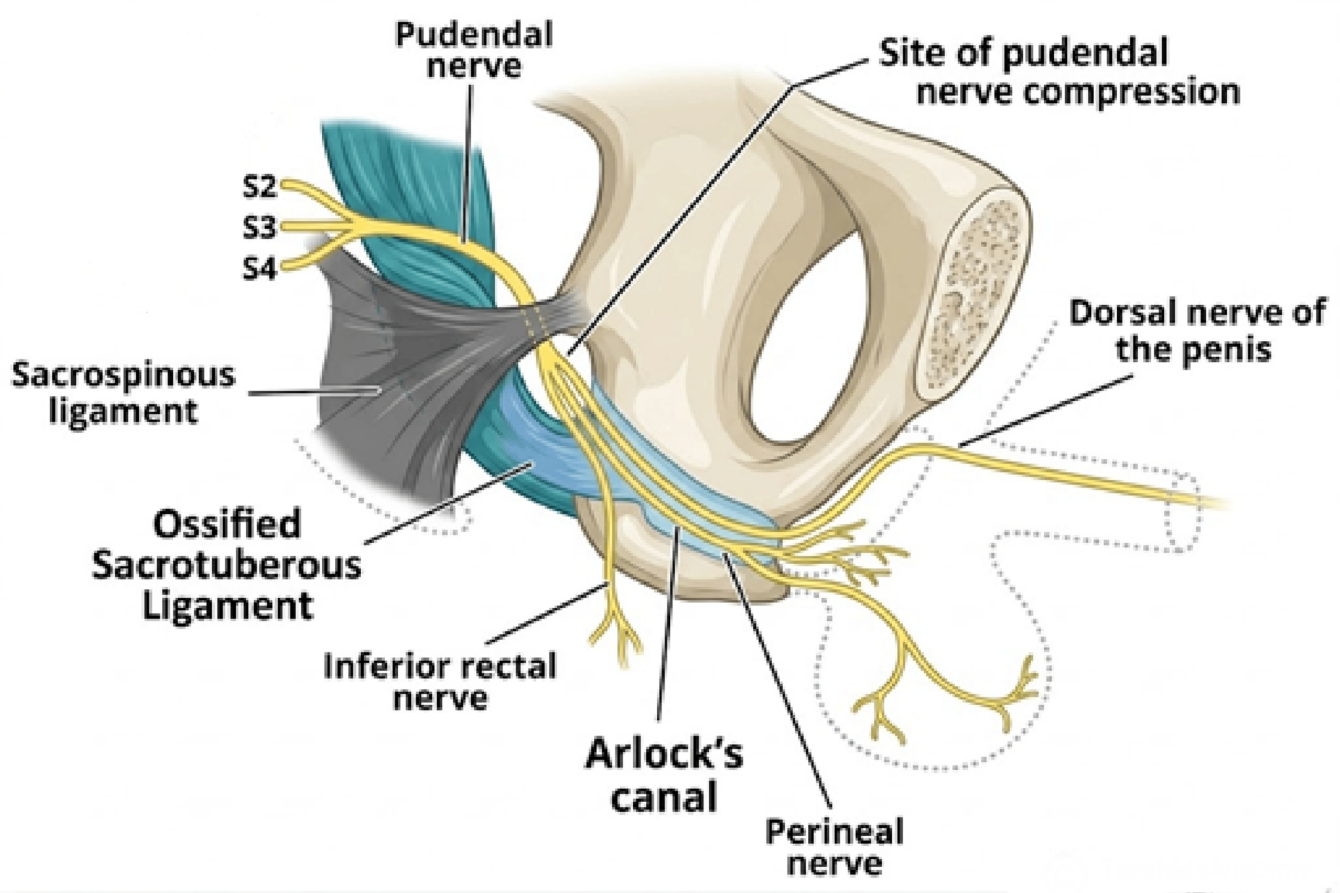
Schematic illustration of pudendal nerve entrapment secondary to ossification of the sacrotuberous ligament The diagram demonstrates the anatomical relationship between the pudendal nerve and the sacrotuberous and sacrospinous ligaments, highlighting the presumed site of pudendal nerve compression caused by ossification of the sacrotuberous ligament during its course toward Alcock's canal. Figure was created by the authors using MS Paint and MS PowerPoint (Microsoft, Redmond, Washington)

A schematic illustration demonstrating the anatomical relationship between the pudendal nerve and the ossified sacrotuberous ligament, including the presumed site of nerve compression, is presented in Figure 3.

The patient was managed conservatively with ultrasound-guided local corticosteroid injections administered at three-month intervals over a one-year period. Ultrasound-guided injections were performed using a 22-gauge spinal needle targeting the region adjacent to the ossified sacrotuberous ligament and presumed pudendal nerve compression site, with administration of 40 mg triamcinolone acetonide diluted in 3 mL of 1% lidocaine. Progressive symptomatic improvement was observed throughout follow-up, with gradual resolution of pain, sensory disturbances, and erectile dysfunction. At the two-year follow-up evaluation, the patient remained asymptomatic, with no evidence of recurrence and marked improvement in functional status and quality of life. No additional interventional or pharmacological therapies were initiated during the treatment period.

A chronological timeline summarizing the patient's clinical presentation, diagnostic evaluation, treatment course, and follow-up outcomes is presented in Table 1. 

**Table 1:** Timeline of clinical presentation, diagnostic evaluation, treatment, and follow-up Chronological summary of the patient’s symptoms, imaging findings, conservative management, and long-term clinical outcome following pudendal nerve entrapment secondary to sacrotuberous ligament ossification.

Time point	Clinical event	Findings/Intervention	Outcome
Initial clinical evaluation	Physical examination	Right-sided hip and perineal pain, burning sensation, foreign body sensation, erectile dysfunction, reduced superficial sensation in the perineal region	Suspicion of pudendal neuropathy
Initial imaging	Pelvic radiography	Partial ossification of the right sacrotuberous ligament was identified	Further imaging recommended
Subsequent imaging	3D computed tomography	Confirmed extent and morphology of STL ossification; no additional pelvic or spinal pathology	Diagnosis supported
Month 0	Initiation of conservative treatment	Ultrasound-guided corticosteroid injection; activity modification; specialized seating cushion	Partial symptom improvement
Months 3-9	Serial follow-up treatment	Repeated ultrasound-guided corticosteroid injections administered at three-month intervals	Progressive reduction in pain, sensory disturbances, and erectile dysfunction
Month 12	Completion of treatment course	Conservative management concluded	Complete resolution of symptoms
2-year follow-up	Long-term evaluation	Clinical reassessment performed	Patient remained asymptomatic without recurrence

## Discussion

The first description of sacrotuberous ligament (STL) ossification was reported by Gruber in 1876 and primarily involved the superior portion of the ligament [5]. Calcification of soft tissues within the musculoskeletal system is a well-recognized phenomenon and, in many cases, represents the late result of a chronic tissue damage-repair process. Such calcific changes may involve a variety of tissues, including synovium, muscle, and cartilage [6].

Pudendal neuralgia is a severely painful and disabling neuropathic pain syndrome affecting both men and women within the sensory distribution of the pudendal nerve. Entrapment neuropathies develop when peripheral nerves are compressed while traversing rigid anatomical boundaries, such as fibro-osseous tunnels or fascial planes [7]. Pudendal nerve entrapment (PNE) is considered an underrecognized clinical entity, as true pudendal nerve entrapment has been identified in only approximately 27% of patients presenting with pudendal neuralgia in a previous cohort study [8].

Anatomically, four principal sites of pudendal nerve entrapment have been described [9]. Among these, entrapment between the sacrospinous and sacrotuberous ligaments (type II entrapment) is by far the most common, accounting for nearly 74% of reported cases [8]. In the present report, we describe a case of pudendal nerve entrapment secondary to ossification of the sacrotuberous ligament.

The sacrotuberous ligament originates from the posterior iliac spines and upper coccygeal region and courses obliquely downward and laterally toward the ischial tuberosity, where it lies posterior to the sacrospinous ligament [1]. The pudendal nerve, derived from the ventral rami of S2-S4, maintains a close anatomical relationship with the STL, passing posterior to the sacrospinous ligament and medial to the sacrotuberous ligament before entering the lesser sciatic foramen [9]. This intimate anatomical relationship explains how structural abnormalities of the sacrotuberous ligament may result in pudendal nerve compression.

Ossification or calcification of the sacrotuberous ligament is an exceptionally rare finding and has been reported predominantly in elderly male patients [10]. The underlying pathophysiological mechanisms are believed to be multifactorial and may include chronic mechanical stress, degenerative changes, excessive mineral intake, post-traumatic or post-surgical repair processes, and, in some cases, suboptimal operative techniques [11,12]. Given the advanced age at which this condition is typically identified, age-related degenerative changes are considered more likely contributors than an idiopathic process.

Progressive calcification or ossification may reduce ligamentous elasticity and increase rigidity, thereby predisposing adjacent neurovascular structures, particularly the pudendal nerve, to mechanical compression. Patients with pudendal nerve entrapment typically present with pain in the perineal, genital, or anorectal region, often exacerbated by prolonged sitting [13]. Neuropathic symptoms such as burning sensations, numbness, paresthesia, or "pins and needles" may also be present. In more advanced cases, urinary, bowel, or sexual dysfunction may coexist, significantly impairing quality of life. Importantly, pudendal nerve entrapment may also manifest as erectile dysfunction in the absence of overt neuropathic pain [14].

Radiologically, ossification or calcification of the STL has been described as a slate pencil-like formation originating from the ischial tuberosity, projecting toward the obturator foramen, and demonstrating a caudocranial growth pattern [10]. Diagnosis may be challenging and is commonly based on clinical examination, including vaginal or rectal palpation, in conjunction with the Nantes criteria [13]. As demonstrated in the present case, imaging modalities are particularly valuable in defining the extent, morphology, and density of STL ossification, while magnetic resonance imaging may further assist in assessing adjacent soft tissues and potential nerve compression. In addition, a diagnostic pudendal nerve block using local anesthetics may help confirm the diagnosis.

Therapeutic options remain limited because of the rarity of this condition. Although surgical excision has been proposed as a potentially definitive treatment capable of providing sustained pain relief, it has been documented in only a single reported case [4]. Most patients described in the literature have been managed conservatively, including treatment with nonsteroidal anti-inflammatory drugs (NSAIDs), physiotherapy, or clinical observation alone [15].

In the present case, the patient was successfully managed conservatively using specialized seating cushions in combination with repeated ultrasound-guided corticosteroid injections administered at three-month intervals over a one-year period. Additional conservative approaches described in the literature include local anesthetic injections and targeted strengthening of the gluteus maximus muscle. Because the sacrotuberous ligament contributes substantially to pelvic stability, surgical resection may carry a risk of postoperative biomechanical imbalance and secondary musculoskeletal complications. Consequently, conservative management should be considered the first-line approach in patients with manageable symptoms and no indication for urgent surgical decompression. Pulsed radiofrequency therapy has also been described as a minimally invasive treatment option in selected patients with pudendal nerve entrapment and may represent an alternative therapeutic strategy when surgical intervention is considered high risk.

Standardized validated symptom scoring instruments were not prospectively recorded, which limited quantitative assessment of symptom severity and treatment response over time.

Although the patient's clinical presentation and imaging findings strongly suggested pudendal nerve entrapment secondary to sacrotuberous ligament ossification, a formal diagnostic pudendal nerve block and complete application of the Nantes criteria were not performed, which limits definitive confirmation of causality.

## Conclusions

Sacrotuberous ligament ossification is a rare but clinically significant cause of pudendal nerve entrapment that may remain underrecognized in patients presenting with chronic perineal pain and associated neuropathic symptoms. Careful clinical assessment combined with appropriate imaging studies is essential for establishing an accurate diagnosis and excluding alternative causes of pelvic pain. This single case suggests that a conservative regimen including ultrasound‑guided corticosteroid injections and seating modifications may be associated with symptomatic improvement in a patient with pudendal nerve entrapment secondary to STL ossification. However, given the absence of a diagnostic nerve block and quantitative outcome measures, these findings should be interpreted cautiously. Conservative management may be considered as an initial approach before surgery, but further cases or a prospective series are needed to confirm efficacy and durability.
